# Implementation of Best Practices in Pancreatic Cancer Care in the Netherlands: A Stepped-Wedge Randomized Clinical Trial

**DOI:** 10.1001/jamasurg.2023.7872

**Published:** 2024-02-14

**Authors:** Tara M. Mackay, Anouk E. J. Latenstein, Simone Augustinus, Lydia G. van der Geest, Auke Bogte, Bert A. Bonsing, Geert A. Cirkel, Lieke Hol, Olivier R. Busch, Marcel den Dulk, Lydi M. J.W. van Driel, Sebastiaan Festen, Derk-Jan A. de Groot, Jan-Willem B. de Groot, Bas Groot Koerkamp, Nadia Haj Mohammad, Joyce T. Haver, Erwin van der Harst, Ignace H. de Hingh, Marjolein Y. V. Homs, Maartje Los, Saskia A. C. Luelmo, Vincent E. de Meijer, Leonie Mekenkamp, I. Quintus Molenaar, Gijs A. Patijn, Rutger Quispel, Tessa E. H. Römkens, Hjalmar C. van Santvoort, Martijn W.J. Stommel, Niels G. Venneman, Robert C. Verdonk, Frederike G. I. van Vilsteren, Judith de Vos-Geelen, C. Henri van Werkhoven, Jeanin E. van Hooft, Casper H. J. van Eijck, Johanna W. Wilmink, Hanneke W. M. van Laarhoven, Marc G. Besselink

**Affiliations:** 1 Amsterdam UMC, location University of Amsterdam, Department of Surgery, Amsterdam, the Netherlands; 2 Cancer Center Amsterdam, the Netherlands; 3 Department of Research and Development, Netherlands Comprehensive Cancer Organisation (IKNL), Utrecht, the Netherlands; 4 Department of Gastroenterology, Regional Academic Cancer Center Utrecht, University Medical Center Utrecht & St. Antonius Hospital Nieuwegein, the Netherlands; 5 Department of Surgery, Leiden University Medical Center, Leiden, the Netherlands; 6 Department of Medical Oncology, Regional Academic Cancer Center Utrecht, University Medical Center Utrecht & St. Antonius Hospital Nieuwegein, the Netherlands; 7 Department of Gastroenterology, Maasstad Hospital, Rotterdam, the Netherlands; 8 Department of Surgery, Maastricht UMC+, Maastricht, the Netherlands; 9 NUTRIM-School of Nutrition and Translational Research in Metabolism, Maastricht University, Maastricht, the Netherlands; 10 Department of General, Visceral and Transplant Surgery, University Hospital Aachen, Germany, the Netherlands; 11 Department of Gastroenterology, Erasmus Medical Center, Rotterdam, the Netherlands; 12 Department of Surgery, OLVG, Amsterdam, the Netherlands; 13 Department of Medical Oncology, University Medical Center Groningen; 14 Department of Medical Oncology, Isala, Zwolle, the Netherlands; 15 Department of Surgery, Erasmus MC, Rotterdam, the Netherlands; 16 Amsterdam UMC, location University of Amsterdam, Department of nutrition and dietetics, Amsterdam, the Netherlands; 17 Department of Surgery, Maasstad Hospital, Rotterdam, the Netherlands; 18 Department of Surgery, Catharina Hospital, Eindhoven, the Netherlands; 19 Department of Medical Oncology, Erasmus MC, Rotterdam, the Netherlands; 20 Department of Medical Oncology, Leiden University Medical Center, Leiden; 21 Department of Surgery, University of Groningen and University Medical Center Groningen, Groningen, the Netherlands; 22 Department of Medical Oncology, Medisch Spectrum Twente, Enschede, the Netherlands; 23 Department of Surgery, Regional Academic Cancer Center Utrecht, University Medical Center Utrecht & St. Antonius Hospital Nieuwegein, the Netherlands; 24 Department of Surgery, Isala, Zwolle, the Netherlands; 25 Department of Gastroenterology, Reinier de Graaf Hospital, Delft, the Netherlands; 26 Department of Gastroenterology, Jeroen Bosch Hospital, Den Bosch, the Netherlands; 27 Julius Center for Health Sciences and primary care, University Medical Center Utrecht, Utrecht University, the Netherlands; 28 Department of Gastroenterology, Leiden University Medical Center, Leiden, the Netherlands; 29 Amsterdam UMC, location University of Amsterdam, Department of Medical Oncology, Amsterdam, the Netherlands

## Abstract

**Importance:**

Implementation of new cancer treatment strategies as recommended by evidence-based guidelines is often slow and suboptimal.

**Objective:**

To improve the implementation of guideline-based best practices in the Netherlands in pancreatic cancer care and assess the impact on survival.

**Design, setting, and participants:**

This multicenter, stepped-wedge cluster randomized trial compared enhanced implementation of best practices with usual care in consecutive patients with all stages of pancreatic cancer. It took place from May 22, 2018 through July 9, 2020. Data were analyzed from April 1, 2022, through February 1, 2023. It included all patients in the Netherlands with pathologically or clinically diagnosed pancreatic ductal adenocarcinoma. This study reports 1-year follow-up (or shorter in case of deceased patients).

**Intervention:**

The 5 best practices included optimal use of perioperative chemotherapy, palliative chemotherapy, pancreatic enzyme replacement therapy (PERT), referral to a dietician, and use of metal stents in patients with biliary obstruction. A 6-week implementation period was completed, in a randomized order, in all 17 Dutch networks for pancreatic cancer care.

**Main Outcomes and Measures:**

The primary outcome was 1-year survival. Secondary outcomes included adherence to best practices and quality of life (European Organisation for Research and Treatment of Cancer [EORTC] global health score).

**Results:**

Overall, 5887 patients with pancreatic cancer (median age, 72.0 [IQR, 64.0-79.0] years; 50% female) were enrolled, 2641 before and 2939 after implementation of best practices (307 during wash-in period). One-year survival was 24% vs 23% (hazard ratio, 0.98, 95% CI, 0.88-1.08). There was no difference in the use of neoadjuvant chemotherapy (11% vs 11%), adjuvant chemotherapy (48% vs 51%), and referral to a dietician (59% vs 63%), while the use of palliative chemotherapy (24% vs 30%; odds ratio [OR], 1.38; 95% CI, 1.10-1.74), PERT (34% vs 45%; OR, 1.64; 95% CI, 1.28-2.11), and metal biliary stents increased (74% vs 83%; OR, 1.78; 95% CI, 1.13-2.80). The EORTC global health score did not improve (area under the curve, 43.9 vs 42.8; median difference, −1.09, 95% CI, −3.05 to 0.94).

**Conclusions and Relevance:**

In this randomized clinical trial, implementation of 5 best practices in pancreatic cancer care did not improve 1-year survival and quality of life. The finding that most patients received no tumor-directed treatment paired with the poor survival highlights the need for more personalized treatment options.

**Trial Registration:**

ClinicalTrials.gov Identifier: NCT03513705

## Key Points

**Question:**

Can enhanced nationwide implementation of guideline-based best practices in the Netherlands in pancreatic cancer care improve 1-year survival?

**Findings:**

In this Dutch multicenter, stepped-wedge randomized clinical trial including 5887 patients, 1-year survival was 24% after implementation of 5 best practices compared with 23% before, and did not significantly differ.

**Meaning:**

The finding that most patients received no tumor-directed treatment paired with the poor survival highlights the need for more personalized treatment options in pancreatic cancer.

## Teaser

This study aims to improve the nationwide implementation of guideline-based best practices in pancreatic cancer care and assess the impact on survival.

## Introduction

The incidence of pancreatic cancer is increasing and is projected to become the second-leading cause of cancer-related death by 2030.^1^ For patients with pancreatic cancer, the best option for 5-year survival is surgical resection combined with chemotherapy. However, most patients (80%) present with metastatic and locally advanced disease for whom palliative chemotherapy is the main treatment option. Despite the introduction of new chemotherapy regimens, FOLFIRINOX (5-fluorouracil, folinic acid, oxaliplatin, and irinotecan) and gemcitabine with nab-paclitaxel, nationwide overall survival (OS) in the US improved from 3 up to 4 months only.^2^

In addition to tumor biology, the lack of improvement of outcomes over time might be related to noncompliance to guidelines, slow uptake of new treatment regimens, and undesired practice variation.^3,4,5^ The nationwide compliance to 5 quality indicators for the Dutch guideline on management of pancreatic cancer demonstrated little to no improvement in the first 6 years after publication.^6^ Furthermore, the implementation of the new chemotherapy regimens was also found to be suboptimal.^7^ The same holds for the use of self-expandable metal stents, as compared with plastic stents, for adequate biliary drainage (center variation: 0% to 77%).^8^ Lastly, exocrine pancreatic insufficiency is currently undertreated (41%)^9^, which is most likely related to impaired survival.^10,11,12,13^

We designed a nationwide stepped-wedge cluster randomized clinical trial, with the goal to improve guideline compliance, reduce undesired practice variation, and improve survival and quality of life (QOL) of patients with pancreatic cancer. Herein, we aimed to implement 5 best practices in all 17 Dutch networks for pancreatic cancer care: improved use of (1) perioperative chemotherapy, (2) palliative chemotherapy, (3) pancreatic enzyme replacement therapy (PERT), (4) referral to a dietician, and (5) metal instead of plastic stents for biliary drainage.

## Methods

### Trial Oversight

The Dutch Pancreatic Cancer Project (PACAP)-1 trial was conducted as a nationwide multicenter stepped-wedge cluster randomized trial with 17 clusters and a 25-month study duration (May 22, 2018, until July 9, 2020). A cluster included 1 pancreatic surgery center with its regional referral network, the 17 clusters together comprised all hospitals in the Netherlands (eFigure 1 in Supplement 1).^14^ Ethical approval was obtained at the Medical Ethical Committee of the Amsterdam UMC (December 18, 2017; W17_454#17.526). This was confirmed and approved by the ethical boards of all participating pancreatic surgery centers. The trial was designed in accordance with the Consolidated Standards of Reporting Trials Extension (CONSORT Extension) reporting guidelines.^15^ No methodological changes were made after trial commencement. The funders of the study had no role in the study design, data collection, data analysis, data interpretation, writing of the report, and decision to publish. Informed consent and a data monitoring committee were not required because only best practices from current literature and guidelines were implemented. Cluster consent of the pancreatic cancer team from each pancreatic surgery center was obtained.^16^ The trial was registered before initiation at ClinicalTrials.gov (NCT03513705). The study protocol has been published previously.^17^

### Data Collection

All data were collected from existing clinical registries, embedded in the infrastructure of the existing PACAP^18,19^: the Netherlands Cancer Registry, the Dutch Pancreatic Cancer Audit,^20^ and Patient Reported Outcome Measures (PROMs).^18^ The Netherlands Cancer Registry and the Dutch Pancreatic Cancer Audit are both mandatory and include all subsequent patients with the diagnosis pancreatic cancer and undergoing pancreatic surgery, whereas PROMs were only collected after obtaining written informed consent.

### Patients

All patients in the Netherlands with pathologically or clinically diagnosed pancreatic ductal adenocarcinoma, all ages and all stages, during the study period were included. The Netherlands Cancer Registry was notified if a patient was diagnosed with cancer by the Dutch Nationwide Pathology Databank and the Dutch National Hospital Care registration. Notifications were verified in electronic patient files by trained registrars. Patients participating in the registration of QOL provided written informed consent for receiving the questionnaire and linking it to clinical data.^18^

### Randomization and Blinding

All clusters started in the control group (current practice) and all crossed over to the intervention group (best practice) in a randomized stepwise manner, including a wash-in phase (eFigure 2 in Supplement 1). The randomization was performed by an independent statistician, with stratification for annual pancreatic resections (more than 45 vs 45 or less). The sequence of randomization was concealed from patients and the investigators until the wash-in period. Patients and health care professionals were not blinded from treatment. As all data were obtained from existing encoded PACAP registries, data entry was performed independently of the study. To avoid contamination of clusters still in the control phase, best practices were not shared with local clinicians before the transfer to the intervention phase.

### Trial Procedures

Current practices were left to the discretion of the health care professionals in the control phase and national protocols remained the same during the study period. A structured 6-week wash-in phase was designed to achieve implementation of best practices. The wash-in phase included monitoring, return visits, providing feedback in combination with education (plus smartphone application), and reminders. Per cluster, a regional pancreatic cancer team played a key role and served as reference for the other hospitals in the network. The pancreatic cancer team included a medical oncologist, a gastroenterologist, and a surgeon, and when possible, a specialized nurse or dietician.

Best practice treatments included the optimal use of (1) perioperative chemotherapy; target was set at 70% of all patients undergoing resection receiving adjuvant chemotherapy, (2) palliative chemotherapy; target at 40% of all patients with metastatic disease, (3) PERT, (4) referral to a dietician, and (5) endoscopic metal stents instead of plastic for biliary drainage. Best practices were selected based on current available literature, multidisciplinary Dutch Pancreatic Cancer Group meetings, and the evaluation of the implementation of the Dutch National Guideline.^5^ Multiple best practices were assessed and only those who had the potential to improve survival, clinical outcomes, and QOL were selected. Additionally, the trial design was used to implement additional improvements in pancreatic cancer care, such as standardized radiology reports; exact definitions are described in the protocol.^17^

### Outcomes

The primary end point was 1-year survival among all patients. Subgroup analysis on 1-year survival was performed in the subgroups of (borderline) patients with resectable, locally advanced, and metastatic pancreatic cancer. Secondary end points included OS, incidence of the 5 implemented best practice interventions, QOL, safety outcomes/evaluation of adequate use of best treatments, additional practices that were evaluated during the trial, sensitivity analysis, and post hoc analysis; see the published protocol (Supplement 2) for a detailed list of the secondary end points.^17^

### Statistical Analysis

For sample size analysis see eMethods 1 in Supplement 1. Outcomes were evaluated before and after wash-in period (ie, current practice vs best practice). Analysis was performed with an intention-to-treat analysis according to the randomization order. Patients diagnosed during the wash-in period were described but excluded from the primary analysis. Missing data on baseline characteristics were imputed by multiple imputation techniques, using the multivariate imputation via chained equations, with 10 dummy sets. Outcome data were not imputed. Patients who were lost to follow-up within 1 year were censored at the date of loss to follow-up. The primary outcome, 1-year survival, was analyzed with mixed-effects Cox proportional hazards regression models using a random intercept for hospital and a random slope on intervention effect for hospital. The analyses were adjusted for (calendar) time, age at diagnosis, and tumor stage at diagnosis using the Union for International Cancer Control tumor/node/metastasis eighth edition (2018) classification and staging system for pancreatic cancer. Secondary, subgroup, and sensitivity analyses were performed as described in the published protocol, in which other statistical analyses details can also be found.^17^ Some necessary minor deviations were made from the study protocol (eMethods 2 in Supplement 1).

Effect estimates with 95% CIs were reported. All *P* values were based on a 2-sided test. *P* values of less than .05 were considered statistically significant. A statistically significant difference in the incidence of the implemented intervention (eg, application of palliative chemotherapy) was considered a successful implementation. Within QOL analysis, a moderate absolute change of 10 points (on a scale between 0 to 100) was considered clinically relevant according to the method by Osoba et al.^21^ All calculations were performed using RStudio version 4.0.3 (The R Project).

## Results

**Figure 1.**
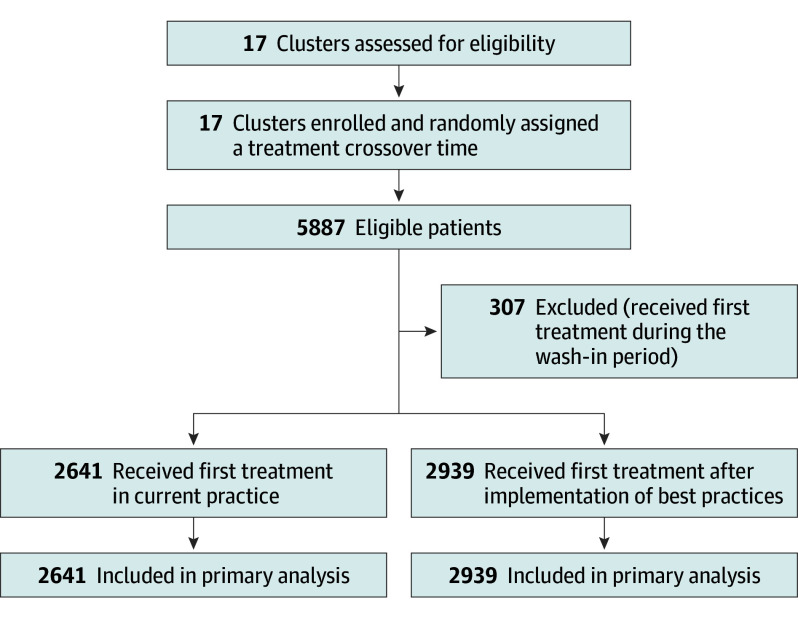
Trial Profile

All 17 networks for pancreatic cancer care were randomized. One center (number 11) stopped performing pancreatic surgery but remained an oncological center for nonresectable disease; therefore, this cluster and the randomization order was not changed. A total of 5887 patients were eligible and included in this study (May 22, 2018, until July 9, 2020), of whom 2641 were in current practice (control group), 307 were in the wash-in phase, and 2939 were in best practice (intervention group; Figure 1). Within all patients, the primary outcome (1-year survival) could be assessed, therefore, there was no loss to follow-up.

### Characteristics of the Patients

Baseline characteristics are provided in Table 1 (wash-in phase in eTable 1 in Supplement 1). Mean age was 72.0 years. Most patients had metastatic disease on diagnosis (3094 [62%]) and a World Health Organization performance status of 2 (37%).

**Table 1.** Baseline Characteristics^a^

Characteristic	No. (%)	
	Current practice (control) (n = 2641)	Best practice (intervention) (n = 2939)
Age, y, median (IQR)	72.0 (64.0-79.0)	72.0 (65.0-79.0)
Sex		
Female	1320 (50)	1483 (50)
Male	1321 (50)	1456 (50)
CCI		
0	1029 (41)	1276 (44)
1	914 (37)	1014 (35)
≥ 2	561 (23)	617 (21)
Missing	137	32
ASA^b^		
1	21 (5.2)	11 (2.8)
2	245 (60)	220 (55)
≥ 3	141 (35)	167 (42)
Missing	8	16
WHO performance status		
0	514 (30)	558 (31)
1	629 (37)	693 (38)
2	310 (18)	286 (16)
≥ 3	247 (15)	276 (15)
Missing	941	1126
Tumor stage (AJCC TNM)		
1A	65 (2.5)	76 (2.6)
1B	239 (9.2)	238 (8.3)
2A	83 (3.2)	95 (3.3)
2B	205 (7.9)	244 (8.5)
3	496 (19)	541 (19)
4	1501 (58)	1677 (58)
Missing	52	68
Resectability		
(Borderline) resectable	653 (28)	792 (29)
LAPC	251 (11)	310 (12)
Metastasized	1457 (62)	1637 (62)
Missing	280	300

Abbreviations: AJCC, American Joint Committee on Cancer; ASA, American Society of Anesthesiologists; CCI, Charlson Comorbidity Index; LAPC, locally advanced pancreatic cancer; TNM, tumor/node/metastasis; WHO, World Health Organization.

^a^ Based on nonimputed data. When missing values are not described, data are complete.

^b^ Only in patients that underwent resection (n = 862, including wash-in phase [n = 33]).

### 1-Year Survival

**Figure 2.**
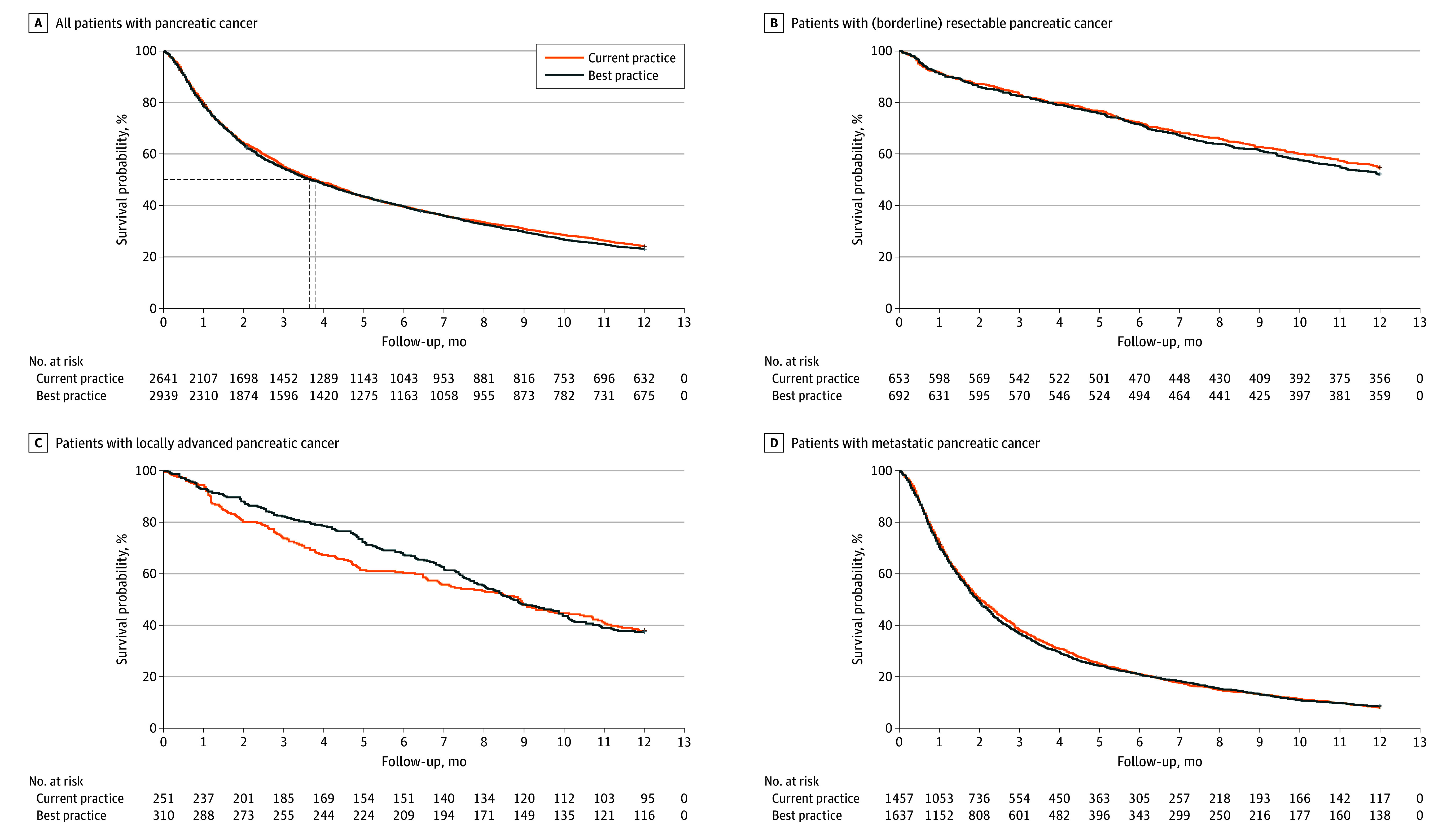
One-Year Overall Survival Among Patient Groups

The primary outcome, 1-year survival, was 24% (634 of 2638) in the control group and 23% (678 of 2939) in the best practice group. The 1-year survival did not change between the control and intervention group (hazard ratio, 0.98; 95% CI, 0.88-1.08; *P* = .66; Table 2). The 1-year survival also did not increase in the subgroups of patients with (borderline) resectable disease, locally advanced, or metastatic pancreatic cancer. Kaplan-Meier curves are shown in Figure 2.

**Table 2.** Overall Survival in Months and 1-Year Survival^a^

Group	No.	Overall survival,^b^ adjusted %		1y Survival, No. (%)		Difference 1-year survival, HR (95% CI)^c^	*P* value
		Current practice	Best practice	Current practice	Best practice		
All patients	5580	3.8	3.7	634 (24.0)	678 (23.1)	0.98 (0.88-1.08)	.66
Subgroups							
(Borderline) resectable pancreatic cancer	1562	13.6	12.2	385 (51.0)	387 (48.0)	1.14 (0.91-1.42)	.26
Locally advanced pancreatic cancer	871	8.9	8.7	127 (31.5)	147 (31.4)	0.93 (0.71-1.22)	.60
Metastatic pancreatic cancer	3147	2.0	1.9	122 (8.2)	144 (8.7)	0.95 (0.86-1.05)	.34

^a^ Median follow-up of patients alive for current practice: 35.9 months, best practice: 24.7 months.

^b^ Outcomes are based on the nonimputed set, no missing data in survival outcomes.

^c^ After imputation of baseline characteristics, no missing data in survival outcomes. The analyses were adjusted for (calendar) time, age at diagnosis, and tumor stage at diagnosis using the Union for International Cancer Control tumor/node/metastasis eighth edition (2018) classification and staging system for pancreatic cancer.

### Adherences to Best Practices

The adherences to the best practices are shown in Table 3. The overall use of chemotherapy, all stages combined, was 33.1% (current practice) vs 36.7% (best practice); odds ratio (OR), 1.17; 95% CI, 0.97-1.42; *P* = .09. No difference in use of neoadjuvant/induction chemotherapy in all patients with nonmetastatic disease could be demonstrated (adjusted values: 11.7% vs 11.6%; OR, 1.19; 95% CI, 0.66-2.12; *P* = .57), as well as in the use of adjuvant therapy after resection (adjusted values: 48.4% vs 52.0%; OR, 1.16, 95% CI, 0.74-1.84; *P* = .51). The use of palliative chemotherapy (adjusted values: 23.9% vs 29.6%; OR, 1.34; 95% CI, 1.06-1.69; *P* = .01), PERT (adjusted values: 34.7% vs 45.0%; OR, 1.60; 95% CI, 1.26-2.04; *P* < .001), and metal biliary stents (73.3% vs 82.3%; OR, 1.76, 95% CI, 1.15-2.69; *P* = .01) increased. There was no statistically significant difference in the referral to a dietician (59.3% vs 62.9%; OR, 1.16; 95% CI, 0.92-1.45; *P* = .21).

**Table 3.** Implementation of Best Practices

Best practice treatments	No.^a^	Adjusted %		Difference in best practice treatments^b^	
		Current practice	Best practice	OR (95% CI)^b^	*P* value
Neoadjuvant chemotherapy in nonmetastasized patients^c^	2433	10.9	11.3	1.31 (0.69-1.45)	.41
Adjuvant chemotherapy in patients after surgical resection^d^	829	48.3	51.2	1.13 (0.71-1.79)	.60
Palliative chemotherapy in patients with metastasized pancreatic cancer	3147	23.9	30.3	1.38 (1.10-1.74)	.01
Pancreatic enzyme replacement therapy	5580	34.2	45.2	1.64 (1.28-2.11)	<.001
Referral to dietician	5580	59.3	62.9	1.16 (0.92-1.45)	.21
Use of metal stents for biliary endoscopic drainage	1725	74.1	83.3	1.78 (1.13-2.80)	.01

Abbreviations: OR, odds ratio; WHO, World Health Organization.

^a^ No. of patients in the subgroup the outcome is evaluated in (after imputation of baseline characteristics), in all outcomes with no missing data, or less than 0.1% is present.

^b^ Using a random intercept for hospital and a random slope on intervention effect for hospital and adjusted for (calendar) time.

^c^ Including patients that received induction chemotherapy only, but did not undergo resection.

^d^ Random intercept removed to mitigate singularity errors of the random effects.

### QOL

QOL was evaluated in 610 patients (10%), of whom 277 were in the control group (45%) and were 308 in the intervention group (50%) (and 25 were in the wash-in phase.) The Global Health Score was comparable among patients in the current and best practice group (area under the curve, 43.8 vs 42.7; mean difference, −1.12; 95% CI, −2.13 to 0.89; *P* = .28). The δ Global Health Score, and the area under the curve of all subdomains of the EORTC Core Quality of Life Questionnaire and the EORTC Quality of Life Questionnaire-Pancreatic Cancer Module were similar in both groups (eTables 3 and 4 in Supplement 1). The δ of the EORTC Core Quality of Life Questionnaire and the EORTC Quality of Life Questionnaire-Pancreatic Cancer Module were significantly different in 3 domains at only 1 time point, but all were not considered clinically relevant (δ < 10).

### Evaluations of Adequate Use

Safety outcomes/evaluations of adequate use of best practices were not different among groups (eTable 5 in Supplement 1). Namely, there was no difference among groups in complications of chemotherapy and patients with metastatic disease who received chemotherapy in the last month of life. Stent-related complications were equal among plastic and metal biliary stents (23% vs 23%).

### Additional Best Practices Evaluated

Additional practices in pancreatic cancer care that were evaluated during the trial are depicted in eTable 5 in Supplement 1. Of these, the only significant difference was that more patients participated in the QOL registration in the intervention group (8.9% vs 12.1%; OR, 1.41; 95% CI, 1.05-1.90; *P* = .02).

### Sensitivity Analysis

In all the predefined sensitivity analysis, no difference in 1-year survival was seen (eTable 6 in Supplement 1). Namely, in the groups (1) before and after the publication of the national guideline, (2) if the current and best practice were defined by the date of last treatment instead of first treatment received, and (3) complete case analysis.

### Post hoc Analysis

Post hoc analysis shows that 61% of patients received no tumor-targeted therapy (eTable 2 in Supplement 1). Within the group who did receive tumor-targeted therapy, OS was higher compared with the whole population, 12 months compared with 4 months.

## Discussion

This first nationwide, stepped-wedge cluster randomized clinical trial of patients with all stages of pancreatic cancer in the Netherlands found no improvement in 1-year survival and QOL, although the use of 3 of 5 best practice treatments increased significantly (ie, palliative chemotherapy, PERT, metal biliary stents). Over 60% of patients received no tumor-directed treatment, which is reflected by the poor 1-year survival of 23%.

The lack of improvement in 1-year survival could be explained in several ways. First, in the PACAP-1 trial, the median OS of all patients was less than 4 months and only 2 months for patients with metastatic disease (this group comprises 62% of the study population). It could be questioned whether it was a realistic scenario to be able to increase 1-year survival in such a patient population. We could have opted to not include patient with metastatic disease to improve survival and the likelihood of treatment. However, we previously identified that this subgroup has the largest proportion of untreated patients.^2^ Since such a study design has not been used before in patients with pancreatic cancer, it was difficult to predict to what extent the use of systemic chemotherapy and other best practices could be improved. Importantly, for other cancers with poor survival, such as esophageal and gastric cancer, it has also been shown that improvements in survival are mainly observed in selected best-case patients, while improvement of survival for the population as a whole is not observed.^22,23^ Moreover, the Netherlands is a small country, even though significant variability in practice is present, this is potentially less variable compared with other (larger) countries.

Second, even though the number of patients who received palliative chemotherapy increased with 6% (from 23.9% to 30.3%), the target (40%) was not reached. Clearly, this increase was too small to impact 1-year survival. The small increase in palliative chemotherapy may be due to the poor condition of patients at the time of diagnosis, rather than poor implementation of guidelines. It should be noted that because OS is limited and disease-related symptoms often lead to a poor performance status, it is quite conceivable that in the process of clinical evaluation and shared decision-making best supportive care is indeed the best option for some patients.^24,25^ Therefore, future research should focus on identifying which patients correctly and incorrectly not receive any chemotherapy and the reasons for this. The value of liquid or tumor biomarkers for predicting the effectivity of different chemotherapy regimens seems essential.^26^

Most importantly, the PACAP-1 trial highlights the very poor population-based survival rates of patients with pancreatic cancer, and thus, emphasizes the profound difference between real-world data and highly selected patients in most randomized trials. For instance, the 2 months’ OS in patients with metastatic disease in our population-based trial is clearly different from the 9 months (gemcitabine-nab paclitaxel) and 11 months (FOLFIRINOX) in recent randomized trials.^27,28^ The OS of patients with (borderline) resectable ranged between 12 to 14 months, which does not differ much from the 16 months (ie, intention-to-treat population) in the recent neoadjuvant PREOPANC trial.^29^ It is, however, very different to recent adjuvant trials (eg, modified FOLFIRINOX; 55 months).^30^ Notably, over 60% of patients in the current trial received no tumor-targeted therapy at all (eTable 1 in Supplement 1), most likely explaining this difference. In fact, median OS was 7 months in the 39% of patients with metastatic disease who did receive tumor-targeted therapy and 32 months for patients with (borderline) resectable disease who underwent resection and received perioperative chemotherapy. OS on a nationwide level in the PACAP trial in the Netherlands is actually comparable with nationwide studies from Denmark (5 months) and Germany (6 months).^31,32^ These differences highlight the importance of additionally informing patients on generalizable population-based data.

Even though the use of PERT (absolute increase 10%) and metal stents (absolute increase 9%) increased, there was no improvement in 1-year survival and QOL. Although PERT is associated with improved survival, this increase will not suffice to make a clinically meaningful impact.^10^ As the referral to a dietician unfortunately did not increase jointly with the use of PERT, there could be an underutilization of the best practice. Also, metal stents, compared with plastic stents, result in longer stent patency, lower complication rates, and fewer reinterventions.^33^ This could potentially have an indirect effect on survival as well, as more patients are eligible for future therapy.^33^ However, also here a 10% increase did not have a sufficient impact. Equal complication rates for plastic and metal stents could be due to selection bias. For example, plastic stents can be shortly placed when awaiting the final diagnosis (short-term) and replaced with metal stents when a final diagnosis is made (long-term). ^34^

No differences were seen in the QOL of patients between both groups, even though QOL is known to be positively affected by all of the 3 best practices.^35,36,37,38^ The PROMs were only registered in a subset of patients (10%) and this may have reduced the power of detecting smaller increases in QOL. Future studies should focus on improving inclusion of patients in PROMS evaluation to enable complete and accurate QOL evaluation. This is emphasized by the fact that QOL outcome measures have become an integral part of cancer research^39^ and is in almost one-third of all patients valued over length of life.^40^

The stepped-wedge approach has previously been used to improve outcomes on a national scale, showing different results.^41^ The EPOCH trial^42^ implemented a quality improvement program in 15 873 patients undergoing emergency open major abdominal surgery in the United Kingdom failed to improve survival. The PORSCH trial^43^ implemented an algorithm for early recognition and detection of postoperative complications on postoperative outcomes in 1805 patients after pancreatic surgery in the Netherlands almost halved 90-day mortality The multicenter HuCare2 trial^44^ successfully improved QOL in 762 patients with cancer in Italy (mostly breast cancer; 5% pancreatic cancer) by implementing a quality improvement strategy. This demonstrates that a large stepped-wedge design is feasible, but it is challenging to achieve desired clinical outcomes on a national scale. This has to be taken into account within future trials.

### Limitations

This study has several limitations. First, decisions on the best practices were based on current national guidelines, literature reviews, and multidisciplinary meetings. However, this could be considered somewhat subjective. Second, all data are collected within the national registries; therefore, some outcomes are only assessed in a subset of patients. This selection had to be made due to financial reasons that could not be covered by the trial budget. Nevertheless, using outcomes from national databases increases generalizability and efficiency, making it possible to include patients on a nationwide level. Third, the social view to pancreatic cancer on a nationwide level in the Netherlands may differ from that in other countries, including a negative view on systemic therapy, higher rates of euthanasia, and increased value of QOL over length of life.^45^ This may have influenced the survival rates, of especially the metastatic patients, although reliable data are lacking thus requiring further study. Fourth, the 1-year follow-up could be too short for the (borderline) resectable group, as the median survival is not reached yet. Therefore, some benefits may have been missed. Long-term results (3 to 5 years) have to be awaited.

## Conclusion

In conclusion, this nationwide stepped-wedge randomized clinical trial did not improve 1-year survival or QOL, although it was successful in enhancing the nationwide implementation of 3 best practices in pancreatic cancer. The poor 1-year survival emphasizes the need for improved patient selection, early detection programs, and personalized treatment to improve nationwide outcomes.

## Notes

Supplement 1. eMethods 1. Sample size analysis eMethods2. Deviations to the protocol eFigure 1. Schematic representation of individual hospitals within the 17 pancreatic cancer networks in the Netherlands eFigure 2. Schematic overview of the stepped-wedge cluster RCT design in the PACAP-1 trial eTable 1. Baseline characteristics including wash-in phase eTable 2. Median overall survival in months in subgroups receiving the best practices treatments eTable 3. Outcomes QLQ-C30 eTable 4. Outcomes QLQ-PAN26 eTable 5. Secondary study endpoints eTable 6. Subgroup- and sensitivity analysis of 1-year survival

Supplement 2. Trial protocol

Supplement 3. Nonauthor collaborators

Supplement 4. Data sharing statement
