# Supplementary material for: Implementation of Best Practices in Pancreatic Cancer Care in the Netherlands: A Stepped-Wedge Randomized Clinical Trial
Source: JAMA Surg. 2024 Feb 14;159(4):429–37. doi: 10.1001/jamasurg.2023.7872 (PMC10867778; doi:10.1001/jamasurg.2023.7872)
Supplement: Supplement 1. — eMethods 1. Sample size analysis eMethods2. Deviations to the protocol eFigure 1. Schematic representation of individual hospitals within the 17 pancreatic cancer networks in the Netherlands eFigure 2. Schematic overview of the stepped-wedge cluster RCT design in the PACAP-1 trial eTable 1. Baseline characteristics including wash-in phase eTable 2. Median overall survival in months in subgroups receiving the best practices treatments eTable 3. Outcomes QLQ-C30 eTable 4. Outcomes QLQ-PAN26 eTable 5. Secondary study endpoints eTable 6. Subgroup- and sensitivity analysis of 1-year survival [file jamasurg-e237872-s001.pdf]

Supplemental Online Content

Mackay TM, Latenstein AEJ, Augustinus S, et al; for the Dutch Pancreatic Cancer Group. Dutch implementation of best practices in pancreatic cancer care: a randomized clinical trial. *JAMA Surg*. Published online February 14, 2024.
doi:10.1001/jamasurg.2023.7872

eMethods 1. Sample size analysis

eMethods 2. Deviations to the protocol

eFigure 1. Schematic representation of individual hospitals within the 17 pancreatic cancer networks in the Netherlands

eFigure 2. Schematic overview of the stepped-wedge cluster RCT design in the PACAP-1 trial

eTable 1. Baseline characteristics including wash-in phase

eTable 2. Median overall survival in months in subgroups receiving the best practices treatments

eTable 3. Outcomes QLQ-C30

eTable 4. Outcomes QLQ-PAN26

eTable 5. Secondary study endpoints

eTable 6. Subgroup- and sensitivity analysis of 1-year survival

This supplemental material has been provided by the authors to give readers additional information about their work.

eMethods 1. Sample size analysis

A sample size was first calculated for the pancreatic surgery centers only, as for practical reasons the same randomization order was used as within the PORSCH trial (which only included the pancreatic surgery centers). A reduction of 10% in 1-year all-cause mortality in pancreatic surgery centers was considered clinically relevant. A 25-month study duration provided 80% statistical power for a mortality reduction of 10.0% in the 17 pancreatic surgery centers with a required sample size of 2142 patients, using a two-sided alpha of 0.05, an intracluster correlation (ICC) of 0.02, and cluster autocorrelation of 1. The same study duration yielded 80% for a 6.6% 1-year mortality reduction with an expected 4769 patients nationwide, assuming the ICC will be higher when including all hospitals (including both the pancreatic surgery and the referral centers).

eMethods 2. Deviations to the protocol

The study protocol described the inclusion of length and weight in the baseline characteristics, due to 80% missing values these are only described in the supplementary material. Additionally, descriptive statistics were planned to use to evaluate the adherence of the best practices. To determine the independent effect of the intervention, mixed-effect linear regression models using a random intercept for hospital and a random slope on intervention effect for hospital and adjusted for (calendar) time were performed instead. Consequently, adjusted percentages (considering the random intercept, -slope, and adjustment for calendar time) were reported. A sensitivity analysis before and after the implementation of the European Society of Gastrointestinal Endoscopy guideline on stenting was planned. This could not be performed, as this guideline was not published within our study period. Post-hoc analysis was performed assessing the number of patients who did not receive tumor targeted therapy, and the OS rates of the patients who received the best practice treatments (perioperative / palliative chemotherapy, and PERT).

© 2024 Mackay TM et al. *JAMA Surgery*.

eFigure 1. Schematic representation of individual hospitals within the 17 pancreatic cancer networks in the Netherlands

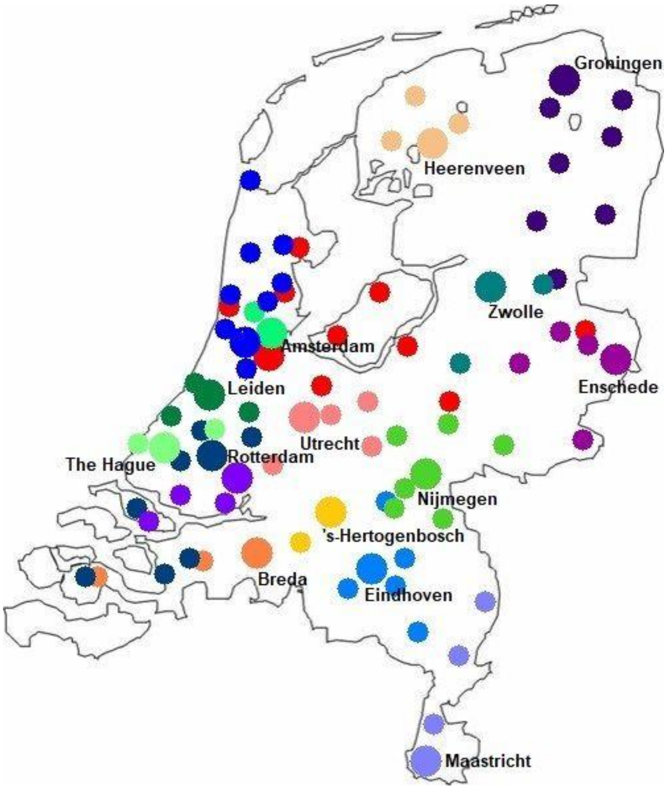

One colour reflects on regional network.
The large dots represent the 17 centers for pancreatic surgery.
The small dots represent the referring hospitals per network.

© 2024 Mackay TM et al. *JAMA Surgery.*

eFigure 2. Schematic overview of the stepped-wedge cluster RCT design in the PACAP-1 trial

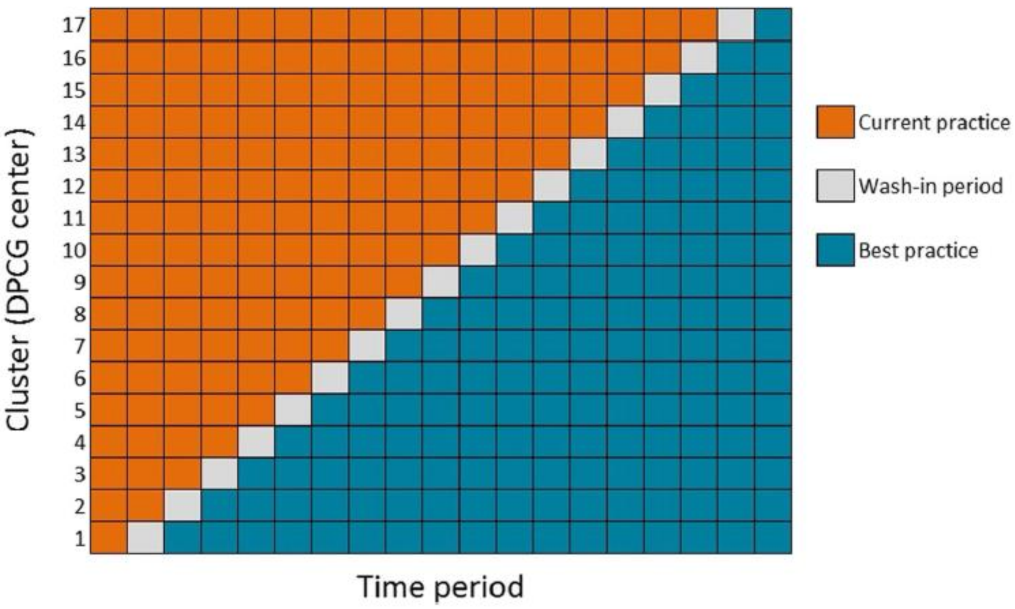

© 2024 Mackay TM et al. *JAMA Surgery*.

eTable 1. Baseline characteristics including wash-in phase

	**Current practice (control) (n = 2641)**	**Wash-in (n= 307)**	**Best practice (intervention) (n =2939)**
Age (median, IQR)	72.0 (64.0-79.0)	72.0 (65.0-78.5)	72.0 (65.0-79.0)
Sex			
Female	1320 (50%)	149 (49%)	1483 (50%)
Male	1321 (50%)	158 (51%)	1456 (50%)
CCI			
0	1029 (41%)	141 (48%)	1276 (44%)
1	914 (37%)	89 (30%)	1014 (35%)
≥ 2	561 (23%)	63 (22%)	617 (21%)
Missing	137	14	32
ASA*			
1	21 (5.2%)	1 (3.1%)	11 (2.8%)
2	245 (60%)	17 (53.%)	220 (55%)
≥ 3	141 (35%)	14 (44%)	167 (42%)
Missing	8 (1.9%)	1 (3.0%)	16 (3.9%)
Weight (median, IQR)	71.0 (62.0-80.0)	70.0 (62.0-81.5)	71.0 (62.0-80.0)
Missing	2131	268	2563
Length (median, IQR)	170.0 (163.0-177.0)	171.0 (163.0-178.0)	169.0 (164.0-178.0)
Missing	2141	270	2563
WHO performance			
status	514 (30%)	59 (30%)	558 (31%)
0	629 (37%)	77 (38%)	693 (38%)
1	310 (18%)	35 (18%)	286 (16%)
2	247 (15%)	29 (14%)	276 (15%)
≥ 3			
	941	107	1126
Missing			
Tumor stage (AJCC			
TNM)	65 (2.5%)	11 (3.6%)	76 (2.6%)
1A	239 (9.2%)	23 (7.6%)	238 (8.3%)
1B	83 (3.2%)	10 (3.3%)	95 (3.3%)
2A	205 (7.9%)	16 (5.3%)	244 (8.5%)
2B	496 (19%)	63 (21%)	541 (19%)
3	1501 (58%)	181 (60%)	1677 (58%)
4			
	52	3	68
Missing			
Resectability**			
(Borderline) resectable	653 (28%)	64 (24%)	792 (29%
LAPC	251 (11%)	29 (11%)	310 (12%)
Metastasized	1457 (62%)	177 (66%)	1637 (62%)
Missing	280	37	300

CCI: Charlson Comorbidity Index**.** ASA: American Society of Anesthesiologist. WHO: World Health Organization. AJCC: American Joint Committee on Cancer. LAPC: Locally Advanced Pancreatic Cancer. Based on non-imputed data, bold numbers indicate statistical significance. When missing values are not described, data is complete. *Only in patients that underwent resection (n=862, including wash-in phase [n=33]). **Resectability was defined according to the DPCG PREOPANC trial criteria.

© 2024 Mackay TM et al. *JAMA Surgery.*

eTable 2. Median overall survival in months in subgroups receiving the best practices treatments

Subgroups	N (%)[#]	Median OS
All patients	5580 (100%)	3.7
No tumor targeted therapy	3386 (61%)	1.7
Tumor targeted therapy	2194 (39%)	12.4
Tumor targeted therapy + enzymes	1488 (27%)	15.0
(Borderline) resectable	1345 (100%)	13.0
No tumor targeted therapy	496 (37%)	4.2
Resection	155 (12%)	15.0
Chemotherapy + resection	481 (36%)	31.7
Chemotherapy + resection + enzymes	437 (54%)	31.6
Locally advanced pancreatic cancer	561 (100%)	8.8
No tumor targeted therapy	238 (42%)	3.4
Chemotherapy + resection	323 (58%)	14.7
Chemotherapy + resection + enzymes	247 (44%)	15.4
Metastasized	3094 (100%)	1.9
No tumor targeted therapy	2245 (73%)	1.3
Tumor targeted therapy	849 (27%)	7.1
Tumor targeted therapy + enzymes	395 (13%)	8.4

Outcomes are based on the non-imputed set, no missing data in survival outcomes. Median follow-up of patients alive for current practice: 35.9 months, best practice: 24.7 months. LAPC: locally advanced pancreatic cancer. [#]Percentage of patients receiving a specific therapy compared to the total (sub)group of patients: i.e. all patients, (borderline) resectable, locally advanced pancreatic cancer, and metastasized.

© 2024 Mackay TM et al. *JAMA Surgery*.

eTable 3. Outcomes QLQ-C30

eTable 3A. Global Health Score: area under the curve (0 to 12 months)

**All patients**	**Current practice (AUC, 95% CI)**	**Best practice (AUC, 95% CI)**	**Difference (AUC, 95% CI)**	**P-value**
Global health score	43.8 (42.5-45.1)	42.7 (41.5-43.9)	-1.12 (-3.13-0.89)	0.277
**Subgroups**				
Resectable	53.7 (51.7-55.8)	51.9 (49.8-53.9)	-1.87 (-4.94-1.19)	0.231
LAPC	39.6 (36.7-42.6)	39.1 (36.8-41.3)	-0.57 (-4.08-2.93)	0.748
Metastasized	25.2 (22.9-27.6)	26.4 (24.4-28.3)	1.17 (-2.33-4.67)	0.511

AUC: Area under the curve. LAPC: Locally Advanced Pancreatic Cancer. AUC: Area under the curve. CI: confidence interval.

eTable 3B. QLQC30 subscales: area under the curve (0-12 months)

**Subscale**	**Current practice (AUC, 95% CI)**	**Best practice (AUC, 95% CI)**	**Difference (AUC, 95% CI)**	**P-value**
Physical functioning	50.2 (48.8-51.6)	50.0 (48.6-51.3)	-0.28 (-2.40-1.85)	0.798
Role functioning	41.9 (39.9-43.7)	40.5 (38.8-42.3)	-1.30 (-4.18-1.57)	0.375
Emotional functioning	49.7 (48.2-51.1)	50.4 (49.1-51.7)	0.68 (-1.45-2.82)	0.531
Cognitive functioning	52.3 (30.0-53.6)	52.3 (51.1-53.6)	0.00 (-2.07-2.07)	0.998
Social functioning	47.6 (46.0-49.2)	46.9 (45.5-48.4)	-0.64 (-3.04-1.77)	0.605
Fatigue	24.1 (22.5-25.7)	24.1 (22.6-25.5)	-0.01 (-2.43-2.39)	0.988
Nausea & vomiting	7.80 (6.72-8.88)	7.01 (6.02-8.00)	-0.80 (-2.43-0.82)	0.332
Pain	16.5 (14.7-18.2)	15.0 (13.3-16.6)	-1.48 (-4.09-1.13)	0.267
Dyspnea	8.77 (7.31-10.2)	8.64 (7.31-9.97)	-0.14 (-2.32-2.03)	0.897
Appetite loss	16.2 (14.4-18.1)	15.9 (14.3-17.6)	-0.26 (-3.05-2.54)	0.858
Constipation	7.19 (5.88-8.48)	7.61 (6.43-8.79)	-0.43 (-1.51-2.37)	0.665
Diarrhea	10.5 (8.83-12.1)	12.3 (10.8-13.7)	1.81 (-0.6-4.26)	0.146
Financial difficulties	4.65 (3.30-6.00)	4.58 (3.35-5.82)	-0.06 (-2.07-1.97)	0.957

AUC: Area under the curve. LAPC: Locally Advanced Pancreatic Cancer. AUC: Area under the curve. CI: confidence interval.

© 2024 Mackay TM et al. *JAMA Surgery.*

eTable 3C. QLQ-C30 QOL subscales: difference in current and best practices of delta (change between baseline and time point)

	0 to 3 months		0 to 6 months		0 to 9 months		0 to 12 months	
**Scale**	**B (95%CI)**	**P-value**	**B (95%CI)**	**P-value**	**B (95%CI)**	**P-value**	**B (95%CI)**	**P-value**
Global health score	-3.85 (-9.69-2.01	0.200	-1.71 (-7.56-4.14)	0.569	-1.29 (-7.14-4.56)	0.694	-4.39 (-10.62-1.82)	0.171
Physical functioning	-3.93 (-9.76-0.91)	0.114	-1.36 (-6.20-3.47)	0.655	2.25 (-2.59-7.08)	0.477	-4.07 (-1-.14-1.98)	0.192
Role functioning	-0.19 (-8.87-8.47)	0.964	0.21 (-8.45-8.89)	0.965	-1.02 (-9.69-7.64)	0.851	-6.54 (-16.61-3.54)	0.209
Emotional functioning	1.67 (-3.04-6.38)	0.489	0.54 (-5.25-4.16)	0.839	-1.34 (-6.05-3.36)	0.642	-2.66 (-8.54-3.22)	0.380
Cognitive functioning	-1.17 (-6.21-3.87)	0.650	2.01 (-3.02-7.05)	0.519	-2.26 (-7.29-2.78)	0.426	-4.82 (-11.04-1.39)	0.133
Social functioning	-2.28 (-9.49-4.93)	0.537	-2.56 (-9.77-4.65)	0.553	-4.34 (-11.55-2.87)	0.345	-5.39 (-12.36-3.57)	0.243
Fatigue	2.27 (-4.98-9.52)	0.541	1.68 (-5.57-9.94)	0.676	-0.55 (-7.80-6.71)	0.885	4.59 (-3.46-12.66)	0.269
Nausea & vomiting	-2.14 (-8.76-4.46)	0.527	-091 (-7.52-5.70)	0.778	-3.48 (-10.09-3.13)	0.351	-3.86 (-10.88-3.17)	0.287
Pain	4.63 (-3.70-12.96)	0.279	-2.59 (-10.92-5.74)	0.552	-1.85 (-10.18-6.49)	0.660	9.85 (1.06-18.64)	**0.031**
Dyspnea	2.68 (-3.49-8.87)	0.396	-0.90 (-7.08-5.28)	0.793	-3.72 (-9.90-2.46)	0.288	2.85 (-4.04-9.74)	0.422
Appetite loss	-3.65 (-13.28-5.97)	0.459	-5.67 (-15.30-3.95)	0.295	-4.17 (-13.79-5.46)	0.471	-5.75 (-16.65-5.15)	0.306
Constipation	-7.38 (-14.56- -0.20)	**0.045**	1.25 (-5.93-8.43)	0.755	-1.96 (-9.14-5.22)	0.616	1.62 (-6.24-9.45)	0.689
Diarrhea	5.56 (-3.05-14.17)	0.208	2.69 (-5.91-11.30)	0.551	2.39 (-6.22-11.00)	0.575	9.19 (-0.37-18.77)	0.063
Financial difficulties	-1.00 (-5.40-3.39)	0.656	-0.22 (-4.61-4.18)	0.925	01.98 (-6.37-2.42)	0.535	1.80 (-4.92-8.53)	0.603

B: beta. CI: confidence interval. Bold numbers indicate statistical significance.

© 2024 Mackay TM et al. *JAMA Surgery.*

eTable 4. Outcomes QLQ-PAN26

eTable 4A. QLQ-PAN26 subscales: area under the curve (0-12 months)

Subscale	Current practice (AUC)	Best practice (AUC)	Difference (AUC)	P-value
Pancreatic pain	15.1 (13.7-16.4)	14.7 (13.4-15.9)	-0.43 (-2.49-1.63)	0.684
Eating related items	16.1 (14.4-17.8)	14.8 (13.2-16.3)	-1.37 (-3.93-1.21)	0.298
Ascites	16.2 (14.4-18.0)	16.5 (14.9-18.1)	0.25 (-2.40-2.90)	0.852
Burden of treatment	19.5 (18.0-20.9)	19.0 (17.6-20.4)	-0.45 (-2.68-1.77)	0.691
Indigestion	15.5 (13.8-17.3)	15.4 (13.8-17.0)	-0.09 (-2.73-2.53)	0.942
Flatulence	22.6 (20.8-24.4)	24.0 (22.4-25.7)	1.48 (-1.20-4.15)	0.279
Cachexia	15.6 (14.1-17.1)	15.4 (14.1-16.7)	-0.14 (-2.36-2.09)	0.904
Hepatic	5.79 (4.89-6.70)	4.74 (3.90-5.57)	-1.05 (-2.39-0.29)	0.124
Altered bowel habit	20.9 (19.2-22.5)	20.2 (18.7-21.6)	-0.68 (-3.07-1.71)	0.575
Body image	14.1 (12.3-15.8)	13.3 (11.8-14.9)	-0.71 (-3.31-1.89)	0.593
Fear of future health	29.4 (27.3-31.5)	29.9 (28.0-31.7)	0.50 (-2.60-3.60)	0.762
Ability to plan the future	23.5 (21.3-25.6)	23.5 (21.6-25.4)	0.09 (-3.06-3.23)	0.957
Health care satisfaction	39.4 (37.4-41.3)	38.3 (36.6-40.0)	-1.06 (-3.92-1.81)	0.470
Sexuality	30.1 (27.5-32.6)	31.6 (29.4-33.9)	1.61 (-2.23-5.44)	0.411

eTable 4B. QLQ-PAN26 subscales: difference in current and best practices of delta (change between baseline and time point)

	0 to 3 months		0 to 6 months		0 to 9 months		0 to 12 months	
**Scale**	**B (95%CI)**	**P-value**	**B (95%CI)**	**P-value**	**B (95%CI)**	**P-value**	**B (95%CI)**	**P-value**
Pancreatic pain	6.42 (-0.02-12.9)	0.052	1.91 (-4.88-8.69)	0.584	-0.24 (-6.72-6.23)	0.694	1.72 (-6.48-9.93)	0.683
Eating related items	2.09 (-6.48-10.68)	0.634	1.72 (-8.05-11.49)	0.732	2.04 (-7.39-11.47)	0.694	1.34 (-10.05-12.73)	0.819
Ascites	-0.08 (-0.47-0.31)	0.694	-4.38 (-12.0-4.3)	0.323	3.76 (-4.94-12.47)	0.400	2.06 (-9.61-13.74)	0.732
Burden of treatment	0.82 (-6.03-7.67)	0.815	1.32 (-6.30-8.94)	0.736	-1.75 (-6.45-9.96)	0.677	8.18 (0.54-15.82)	**0.039**
Indigestion	6.06 (-1.83-13.94)	0.135	1.17 (-7.57-9.92)	0.796	4.59 (-4.62-13.81)	0.694	7.28 (-2.49-17.04)	0.149
Flatulence	-0.08 (-0.47-0.31)	0.694	3.69 (-5.32-12.70)	0.425	6.53 (-3.59-16.65)	0.209	3.19 (-6.28-12.65)	0.514
Cachexia	1.48 (-5.00-7.96)	0.657	-0.39 (-7.95-7.16)	0.919	-3.39 (-12.2-5.39)	0.452	1.74 (-6.26-9.73)	0.673
Hepatic	-2.24 (-9.19-4.71)	0.529	-3.66 (-11.83-4.52)	0.383	-6.18 (-15.39-3.04)	0.192	-6.25 (-16.22-3.74)	0.224
Altered bowel habit	4.04 (-3.13-11.94)	0.255	-1.84 (-9.89-6.22)	0.656	-0.09 (-8.89-8.71)	0.984	-3.05 (-12.3-6.25)	0.524
Body image	-3.05 (12.34-6.25)	0.524	-3.02 (-9.63-3.59)	0.373	-1.29 (-9.53-6.93)	0.758	-7.29 (-15.14-0.55)	0.071

© 2024 Mackay TM et al. *JAMA Surgery.*

Fear of future health	-2.06 (-9.95-5.83)	0.611	-1.19 (-10.05-7.66)	0.793	2.40(-7.19-12.00)	0.626	5.46 (-4.80-15.72)	0.302
Ability to plan the future	-6.74 (16.24-2.77)	0.167	0.63 (-10.24-11.49)	0.910	-1.12 (-12.22-9.98)	0.8440	-0.12 (-12.61-12.37)	0.985
Health care satisfaction	3.55 (-5.31-12.41)	0.435	3.59 (-6.70-13.84)	0.498	-2.07 (-14.82-10.67)	0.752	-5.42 (-17.38-6.54)	0.379
Sexuality	10.71 (-0.43-21.96)	0.061	1.59 (-9.30-12.48)	0.777	1.15 (-10.74-13.03)	0.851	-6.97 (-20.48-6.54)	0.317

© 2024 Mackay TM et al. *JAMA Surgery.*

eTable 5. Secondary study endpoints

Best practice registration	N[#] (% missing)	Adjusted %		Difference between current and best practice	
		Current practice	Best practice	OR (95% CI)	P value
Checklist for radiology reports	761[$] (5.4%)	63.8%	64.2%	1.62 (0.44-5.89)	0.466
Standardized tables with intra-operative events in operation report	761[$] (3.2%)	68.7%	76.8%	2.90 (0.66-12.77)	0.159
Standardized tables with complications of surgical treatment in discharge letters	761[$] (4.1%)	75.9%	75.2%	5.05 (0.16-162.31)	0.360
Standard for synoptic reporting pancreatic cancer pathology	761[$] (2.6%)	90.9%	96.0%	8.18 (0.45-85.04)	0.173
Reporting of WHO performance status in (suspected) metastatic and LAPC patients	4018 (0%)	65.2%	62.1%	0.98 (0.77-1.25)	0.888
**Trial participation**					
Inclusion in PACAP PROMs registry	5580 (0%)	8.9%	12.1%	1.41 (1.05-1.90)	**0.024**
Participation in PACAP PancreasParel biobank	761[$] (84.4%)	90.1%	96.1%	6.18 (0.45-85.04)	0.642
Participation in DPCG RCTs*	5580 (0%)	4.6%	4.9%	1.36 (0.81-2.29)	0.246
**Safety outcomes / complications**					
Metastasized patients who started chemotherapy and received this in last month of life**	692 (23.6%)[a]	23.1%	28.1%	1.20 (0.78-1.26)	0.309
Complications chemotherapy (grade 3-4)	2038[b] (55.1%)	50.7%	43.5%	0.73 (0.46-1.15)	0.176
Type of complications<br>Hematological/immune system<br>Gastrointestinal<br>Neurological<br>Other	440[c] (6.6%)	 112 (25.5%)<br>148 (33.6%)<br>18 (4.1%)<br>133 (30.2%)		NA	
Complications metal stents[c]	299 (3.7%)	65 (22.6%)		NA	
Complications plastic stents[c]	107 (2.8%)	24 (23.1%)		NA	
**Locally advanced pancreatic cancer**					
Pathologic confirmation in patients with (suspected) metastatic and LAPC	4018 (0%)	66.3%	65.5%	0.98 (0.74-1.29)	0.868
LAPC patients discussed in regional MDT meeting (diagnostic period)	871 (<0.1%)	81.0%	75.9%	0.73 (0.42-1.25)	0.248
LAPC patients discussed in MDT meeting (2 months after start chemotherapy)	408 (<0.1%)	45.9%	58.5%	1.68 (0.89-3.15)	0.105
LAPC patients who underwent pancreatic resection*	871 (0%)	8.0%	10.2%	1.32 (0.59-3.14)	0.524
LAPC patients who underwent resection after chemotherapy	408 (0%)	14.5%	18.6%	1.39 (0.51-3.78)	0.515
**Additional outcomes**					

© 2024 Mackay TM et al. *JAMA Surgery.*

Users of smartphone application per week[e]	2 (IQR: 1-5)

Bold numbers indicate statistical significance. OR: Odds ratio. CI: confidence interval. WHO: World Health Organisation. Locally Advanced Pancreatic Cancer. MDT: Muti-Disciplinary Team. NA: Not Applicable. LAPC: [a]Number depicts number of patients that received chemotherapy, last date chemotherapy received only evaluated in 2018, attributing to the missing values. [b]Number depicts number of patients that received chemotherapy, 55% missing values on whether there were any complications, as this was only registered for patients within the PACAP PROMs. [c]Evaluated in patients in which a complication was registered during the study period [c]Evaluated in surgical patients registered in Dutch Pancreatic Cancer Audit that received either a plastic or metal stent. [e]Evaluated between 6-08-2018 until 17-11-2019, smartphone application was introduced to aid the physicians in the use of the best pratices. *Only evaluated the trials: PREOPANC (Netherlands Trial Register: 3709), PELICAN (ClinicalTrials.gov Identifier: NCT03690323), IMPALA (Netherlands Trial Register: 4079/4230), HALO, CROSS FIRE (ClinicalTrials.gov Identifier: NCT02791503). *Random intercept removed, to mitigate singularity errors of the random effects [$]Evaluated in the Dutch Pancreatic Cancer Audit (surgical patients only).

© 2024 Mackay TM et al. *JAMA Surgery*.

eTable 6. Subgroup- and sensitivity analysis of 1-year survival

Subgroup	N#	HR (95% CI)	P-value
Low volume (≤ 40 PDs per year)	2577	0.92 (0.79-1.06)	0.256
High volume (>40 PDs per year)	3003	1.07 (0.94-1.20)	0.308
Treated in referring center*	2964	0.98 (0.88-1.09)	0.775
Treated in pancreatic surgery center*	2616	0.89 (0.75-1.05)	0.167
Participating in DPCG prospective trial**	285	1.67 (0.89-3.4)	0.105
**Sensitivity analysis**			
Before new national guideline***	2649	0.95 (0.82-1.09)	0.463
After new national guideline***	2931	1.03 (0.89-1.19)	0.729
Current or best practice defined by date of last treatment	5516	1.03 (0.93-1.13)	0.605
Complete case analysis	2457##	0.99 (0.90-1.10)	0.900

DPCG: Dutch Pancreatic Cancer Group. PD: pancreatoduodenectomy. *If a patient did not receive treatment, it is the center of diagnosis. **Only evaluated the trials: PREOPANC (Netherlands Trial Register: 3709), PELICAN (ClinicalTrials.gov Identifier: NCT03690323), IMPALA (Netherlands Trial Register: 4079/4230), HALO (ClinicalTirals.gov Identifier: NCT02715804), CROSS FIRE (ClinicalTrials.gov Identifier: NCT02791503). ***Published 03-06-2019. #After imputation of baseline characteristics, no missing data in survival outcomes. ##No imputation of baseline characteristics, as this is complete case analysis (n=120 excluded due to missing values).

© 2024 Mackay TM et al. *JAMA Surgery*.
