# Supplementary material for: Implementation of Best Practices in Pancreatic Cancer Care in the Netherlands: A Stepped-Wedge Randomized Clinical Trial
Source: JAMA Surg. 2024 Feb 14;159(4):429–37. doi: 10.1001/jamasurg.2023.7872 (PMC10867778; doi:10.1001/jamasurg.2023.7872)
Supplement: Supplement 3. — Nonauthor collaborators [file jamasurg-e237872-s003.pdf]

Supplemental Online Content: Nonauthor Collaborators
*First name, last name, and suffix (if applicable) are required and will appear in PubMed.

***Group Name(s): The Dutch Pancreatic Cancer Group**							
***First Name and Middle Initial(s)**	***Last Name**	***Suffix (eg, Jr, III)**	Academic Degrees	Institution	Location (city, state/province, country)	Role or Contribution, eg, chair, principal investigator	Group (if more than 1 Group listed in the byline) and/or Subgroup (eg, Steering Committee)
Hendrik	Bos	6	MD PhD	Department of medical oncology, Tjongerschans Hospital	Heereveen	Coauthor	
Koop	Bosscha	7	MD PhD	Department of Surgery, Jeroen Bosch Hospital	Den Bosch	Coauthor	
Lodewijk A. A.	Brosens	8.9	MD PhD	Department of pathology, University Medical Center Utrecht. Department of pathology, Radboud University.	Utrecht, Nijmegen	Coauthor	
Geert-Jan	Creemers	12	MD PhD	Department of medical oncology, Catherina Hospital	Eindhoven	Coauthor	
Wouter L.	Curvers	13	MD PhD	Department of gastroeneterology, Catharina Hospital	Eindhoven	Coauthor	
Sarah	Derks	14.2	MD PhD	Amsterdam UMC, location Vrije Universiteit, department of medical oncology	Amsterdam	Coauthor	
Susan	van Dieren	1.2	PhD Msc	Amsterdam UMC, location University of Amsterdam, department of surgerey	Amsterdam	Coauthor	
Erwin Jan M.	van Geenen	20	MD PhD	Department of gastroeneterology, Radboud UMC	Nijmegen	Coauthor	
Brigitte C.M.	Haberkorn	24	MD	Department of medical oncology, Maasstad Hospital	Rotterdam	Coauthor	
G.J. Maarten	Hemmink	27	MD PhD	Department of gastroenterology, Isala	Zolle	Coauthor	
Chantal	Hoge	29	MD	Department of gastroenterology, Maastricht UMC+	Maastricht	Coauthor	
Akin	Inderson	31	MD PhD	Department of gastroenterology, Leiden University Medical Center	Leiden	Coauthor	

Supplemental Online Content: Nonauthor Collaborators
*First name, last name, and suffix (if applicable) are required and will appear in PubMed.

***First Name and Middle Initial(s)**	***Last Name**	***Suffix (eg, Jr, III)**	Academic Degrees	Institution	Location (city, state/province, country)	Role or Contribution, eg, chair, principal investigator	Group (if more than 1 Group listed in the byline) and/or Subgroup (eg, Steering Committee)
Maarten A.J.M.	Jacobs	32.2	MD PhD	Amsterdam UMC, location Vrije Universiteit, department of gastroenterology	Amsterdam	Coauthor	
Emile D.	Kerver	33	MD	Department of medical oncology, OLVG	Amsterdam	Coauthor	
Mike S.L.	Liem	34	MD PhD	Department of Surgery, Medisch Spectrum Twente	Enschede	Coauthor	
Hans	Lubbinge	35	MD PhD	Department of gastroenterology, Tjongerschans Hospital	Heereveen	Coauthor	
Jennifer M.J.	Schreinemakers	43	MD PhD	Department of surgery, Amphia Hospital	Breda	Coauthor	
Heidi	Schut	44	MD	Department of medical oncology, Jeroen Bosch Hospital	Den Bosch	Coauthor	
Tom	Seerden	45	MD PhD	Department of gastroneterology, Amphia hospital	Breda	Coauthor	
Joanne	Verheij	48.2	MD PhD	Amsterdam UMC, location University of Amsterdam, department of pathology	Amsterdam	Coauthor	
Annelie	Vulink	51	MD	Department of medical oncology, Jeroen Bosch Hospital	Den Bosch	Coauthor	
Caroline A.	Wientjes	52	MD	Department of gastroenterology, OLVG	Amsterdam	Coauthor	
Fennie	Wit	53	MD	Department of surgery, Tjongerschans Hopistal	Heereveen	Coauthor	
Frank J.	Wessels	54	MD PhD	Department of radiology, Regional Academic Cancer Center Utrecht, University Medical Center Utrecht & St. Antonius Hospital Nieuwegein	Utrecht, Nieuwegein	Coauthor	

Supplemental Online Content: Nonauthor Collaborators
*First name, last name, and suffix (if applicable) are required and will appear in PubMed.

***First Name and Middle Initial(s)**	***Last Name**	***Suffix (eg, Jr, III)**	Academic Degrees	Institution	Location (city, state/province, country)	Role or Contribution, eg, chair, principal investigator	Group (if more than 1 Group listed in the byline) and/or Subgroup (eg, Steering Committee)
Babs	Zonderhuis	55.2	MD	Amsterdam UMC, location Vrije Universiteit, department of surgery	Amsterdam	Coauthor	
