# Supplementary material for: Implementation of Best Practices in Pancreatic Cancer Care in the Netherlands: A Stepped-Wedge Randomized Clinical Trial
Source: JAMA Surg. 2024 Feb 14;159(4):429–37. doi: 10.1001/jamasurg.2023.7872 (PMC10867778; doi:10.1001/jamasurg.2023.7872)
Supplement: Supplement 4. — Data sharing statement [file jamasurg-e237872-s004.pdf]

Data Sharing Statement

Mackay. Implementation of Best Practices in Pancreatic Cancer Care in the Netherlands. *JAMA Surg*. Published February 14, 2024. doi:10.1001/jamasurg.2023.7872

Data
Data available: Yes
Data types: Deidentified participant data
How to access data: Data will be send upon reasonable request to the corresponding author.
When available: With publication

Supporting Documents
Document types: Statistical/analytic code, Informed consent form
How to access documents: Data will be send through a secure database upon reasonable request to the corresponding author.
When available: With publication

Additional Information
Who can access the data: Data will be send by anyonone requestion the data, upon reasonable request to the corresponding author.
Types of analyses: For any purpose
Mechanisms of data availability: With a signed data access agreement
